# Coronavirus Disease 2019 (COVID-19): Emerging and Future Challenges for Dental and Oral Medicine

**DOI:** 10.1177/0022034520914246

**Published:** 2020-03-12

**Authors:** L. Meng, F. Hua, Z. Bian

**Affiliations:** 1 The State Key Laboratory Breeding Base of Basic Science of Stomatology (Hubei-MOST) and Key Laboratory of Oral Biomedicine Ministry of Education, School and Hospital of Stomatology, Wuhan University, Wuhan, China; 2 Center for Evidence-Based Stomatology, School and Hospital of Stomatology, Wuhan University, Wuhan, China

**Keywords:** virology, infection control, dental public health, dental education, transmission, dental practice management

## Abstract

The epidemic of coronavirus disease 2019 (COVID-19), originating in Wuhan, China, has become a major public health challenge for not only China but also countries around the world. The World Health Organization announced that the outbreaks of the novel coronavirus have constituted a public health emergency of international concern. As of February 26, 2020, COVID-19 has been recognized in 34 countries, with a total of 80,239 laboratory-confirmed cases and 2,700 deaths. Infection control measures are necessary to prevent the virus from further spreading and to help control the epidemic situation. Due to the characteristics of dental settings, the risk of cross infection can be high between patients and dental practitioners. For dental practices and hospitals in areas that are (potentially) affected with COVID-19, strict and effective infection control protocols are urgently needed. This article, based on our experience and relevant guidelines and research, introduces essential knowledge about COVID-19 and nosocomial infection in dental settings and provides recommended management protocols for dental practitioners and students in (potentially) affected areas.

## Introduction

On January 8, 2020, a novel coronavirus was officially announced as the causative pathogen of COVID-19 by the Chinese Center for Disease Control and Prevention (Li et al. 2020). The epidemics of coronavirus disease 2019 (COVID-19) started from Wuhan, China, last December and have become a major challenging public health problem for not only China but also countries around the world (Phelan et al. 2020). On January 30, 2020, the World Health Organization (WHO) announced that this outbreak had constituted a public health emergency of international concern (Mahase 2020). The novel coronavirus was initially named 2019-nCoV and officially as severe acute respiratory syndrome coronavirus 2 (SARS-CoV-2). As of February 26, COVID-19 has been recognized in 34 countries, with a total of 80,239 laboratory-confirmed cases and 2,700 deaths (WHO 2020b).

Due to the characteristics of dental settings, the risk of cross infection may be high between dental practitioners and patients. For dental practices and hospitals in countries/regions that are (potentially) affected with COVID-19, strict and effective infection control protocols are urgently needed. This article, based on our experience and relevant guidelines and research, introduces the essential knowledge about COVID-19 and nosocomial infection in dental settings and provides recommended management protocols for dental practitioners and students in (potentially) affected areas.

## What Is COVID-19?

### Viral Etiology

According to recent research, similar to SARS-CoV and Middle East respiratory syndrome coronavirus (MERS-CoV), SARS-CoV-2 is zoonotic, with Chinese horseshoe bats (*Rhinolophus sinicus*) being the most probable origin (Chan et al. 2020; Lu et al. 2020) and pangolins as the most likely intermediate host (The Chinese Preventive Medicine Association 2020).

### Epidemiologic Characteristics

#### Mode of Transmission

Based on findings of genetic and epidemiologic research, it appears that the COVID-19 outbreak started with a single animal-to-human transmission, followed by sustained human-to-human spread (Chan et al. 2020; Del Rio and Malani 2020). It is now believed that its interpersonal transmission occurs mainly via respiratory droplets and contact transmission (The Chinese Preventive Medicine Association 2020). In addition, there may be risk of fecal-oral transmission, as researchers have identified SARS-CoV-2 in the stool of patients from China and the United States (Holshue et al. 2020). However, whether SARS-CoV-2 can be spread through aerosols or vertical transmission (from mothers to their newborns) is yet to be confirmed (Chen, Guo, et al. 2020; WHO 2020c; Zhu et al. 2020).

#### Source of Transmission

Although patients with symptomatic COVID-19 have been the main source of transmission, recent observations suggest that asymptomatic patients and patients in their incubation period are also carriers of SARS-CoV-2 (Chan et al. 2020; Rothe et al. 2020). This epidemiologic feature of COVID-19 has made its control extremely challenging, as it is difficult to identify and quarantine these patients in time, which can result in an accumulation of SARS-CoV-2 in communities (The Chinese Preventive Medicine Association 2020). In addition, it remains to be proved whether patients in the recovering phase are a potential source of transmission (Rothe et al. 2020).

#### Incubation Period

The incubation period of COVID-19 has been estimated at 5 to 6 d on average, but there is evidence that it could be as long as 14 d, which is now the commonly adopted duration for medical observation and quarantine of (potentially) exposed persons (Backer et al. 2020; Li et al. 2020).

#### Fatality Rate

According to current data, the fatality rate (cumulative deaths divided by cumulative cases) of COVID-19 is 0.39% to 4.05%, depending on different regions of China, which is lower than that of SARS (severe acute respiratory syndrome; ≈10%) and MERS (Middle East respiratory syndrome; ≈34% (Malik et al. 2020) and higher than that of seasonal influenza (0.01% to 0.17%) according to data for 2010 to 2017 from the US Centers for Disease Control and Prevention (2020).

#### People at High Risk of Infection

Current observations suggest that people of all ages are generally susceptible to this new infectious disease. However, those who are in close contact with patients with symptomatic and asymptomatic COVID-19, including health care workers and other patients in the hospital, are at higher risk of SARS-CoV-2 infection. In the early stage of the epidemic, in an analysis of 138 hospitalized patients with COVID-19 in Wuhan, 57 (41%) were presumed to have been infected in hospital, including 40 (29%) health care workers and 17 (12%) patients hospitalized for other reasons (Wang et al. 2020). As of February 14, 2020, a total of 1,716 health care workers in China were infected with SARS-CoV-2, consisting of 3.8% affected patients nationally, 6 of that group who have died.

### Clinical Manifestations

The majority of patients with COVID-19 represent relatively mild cases. According to recent studies (Guan et al. 2020; Yang et al. 2020) and data from the National Health Commission of China (2020b), the proportion of severe cases among all patients with COVID-19 in China was around 15% to 25%.

The majority of patients experienced fever and dry cough, while some also had shortness of breath, fatigue, and other atypical symptoms, such as muscle pain, confusion, headache, sore throat, diarrhea, and vomiting (Chen, Zhou, et al. 2020; Guan et al. 2020). Among patients who underwent chest computed tomography (CT), most showed bilateral pneumonia, with ground-glass opacity and bilateral patchy shadows being the most common patterns (Guan et al. 2020; Wang et al. 2020).

Among hospitalized patients in Wuhan, around one-fourth to one-third developed serious complications, such as acute respiratory distress syndrome, arrhythmia, and shock, and were therefore transferred to the intensive care unit (Chen, Zhou, et al. 2020; Huang et al. 2020; Wang et al. 2020). In general, older age and the existence of underlying comorbidities (e.g., diabetes, hypertension, and cardiovascular disease) were associated with poorer prognosis (Kui et al. 2020; Wang et al. 2020; Yang et al. 2020).

### Diagnosis and Treatment

The diagnosis of COVID-19 can be based on a combination of epidemiologic information (e.g., a history of travel to or residence in affected region 14 d prior to symptom onset), clinical symptoms, CT imaging findings, and laboratory tests (e.g., reverse transcriptase polymerase chain reaction [RT-PCR] tests on respiratory tract specimens) according to standards of either the WHO (2020a) or the National Health Commission of China (2020a). It should be mentioned that a single negative RT-PCR test result from suspected patients does not exclude infection. Clinically, we should be alert of patients with an epidemiologic history, COVID-19–related symptoms, and/or positive CT imaging results.

So far, there has been no evidence from randomized controlled trials to recommend any specific anti-nCoV treatment, so the management of COVID-19 has been largely supportive (WHO 2020a). Currently, the approach to COVID-19 is to control the source of infection; use infection prevention and control measures to lower the risk of transmission; and provide early diagnosis, isolation, and supportive care for affected patients (Wang et al. 2020). A series of clinical trials are being carried out to investigate interventions that are potentially more effective (e.g., lopinavir, remdesivir; Del Rio and Malani 2020).

## Infection Control in Dental Settings

### Risk of Nosocomial Infection in Dental Settings

Dental patients who cough, sneeze, or receive dental treatment including the use of a high-speed handpiece or ultrasonic instruments make their secretions, saliva, or blood aerosolize to the surroundings. Dental apparatus could be contaminated with various pathogenic microorganisms after use or become exposed to a contaminated clinic environment. Thereafter, infections can occur through the puncture of sharp instruments or direct contact between mucous membranes and contaminated hands (Kohn et al. 2003).

Due to the unique characteristics of dental procedures where a large number of droplets and aerosols could be generated, the standard protective measures in daily clinical work are not effective enough to prevent the spread of COVID-19, especially when patients are in the incubation period, are unaware they are infected, or choose to conceal their infection.

### Effective Infection Control Protocols

Hand hygiene has been considered the most critical measure for reducing the risk of transmitting microorganism to patients (Larson et al. 2000). SARS-CoV-2 can persist on surfaces for a few hours or up to several days, depending on the type of surface, the temperature, or the humidity of the environment (WHO 2020c). This reinforces the need for good hand hygiene and the importance of thorough disinfection of all surfaces within the dental clinic. The use of personal protective equipment, including masks, gloves, gowns, and goggles or face shields, is recommended to protect skin and mucosa from (potentially) infected blood or secretion. As respiratory droplets are the main route of SARS-CoV-2 transmission, particulate respirators (e.g., N-95 masks authenticated by the National Institute for Occupational Safety and Health or FFP2-standard masks set by the European Union) are recommended for routine dental practice.

## Recommended Measures during the COVID-19 Outbreak

### Recommendations for Management

In January 2020, the National Health Commission of China added COVID-19 to the category of group B infectious diseases, which includes SARS and highly pathogenic avian influenza. However, it also suggested that all health care workers use protection measures similar to those indicated for group A infections—a category reserved for extremely infectious pathogens, such as cholera and plague.

Since then, in most cities of the mainland of China, only dental emergency cases have been treated when strict implementation of infection prevention and control measures are recommended. Routine dental practices have been suspended until further notification according to the situation of epidemics.

Additionally, dentistry-related quality control centers and professional societies in many provinces and cities have put forward their recommendations for dental services during the COVID-19 outbreak, which, as supplementary measures, should be helpful in ensuring the quality of infection control (Li and Meng 2020).

### Current Status of Our School and Hospital

**Figure 1.**
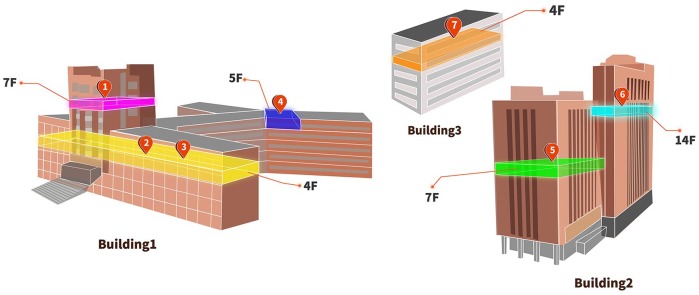
Location of staff and student confirmed with coronavirus disease 2019 (COVID-19) in the main buildings in the School and Hospital of Stomatology, Wuhan University. There are 3 main buildings in our hospital. Building 1 mainly contains outpatients, classrooms, and a library. Its air renewal system depends on air conditioners. Building 2 contains outpatients, wards, and administrative regions. This building was equipped with central air conditioners and a fresh air system. Building 3 is a research laboratory, and it also depends on air conditioners. See Table for details.

The School and Hospital of Stomatology, Wuhan University provided dental care (including oral and maxillofacial surgery) to around 890,000 patients last year and is home to 1,098 staff and 828 students. Our hospital does not have a fever clinic or belong to a designated one for patients with COVID-19. Any staff member who has fever, cough, sneezing, or COVID-19–related symptoms or has a close family member who is confirmed with the infection is advised to undergo a medical examination in a designated hospital and cease working. Since this epidemic, 9 of our colleagues have been confirmed to have COVID-19, including 3 doctors, 3 nurses, 2 administrative staff, and 1 postgraduate student (Fig. 1, Table). So far, there have been no further cases among colleagues or patients who had close contact with them. According to analyses of epidemiologic investigation and medical history, all these cases are without obvious aggregation, except 2 nurses from the same department (patients 2 and 3), and are unlikely to result from cross infection. The infection was possibly limited because medical masks and gloves worn during routine clinic work of dental practitioners prevented further transmission.

**Table.** A Brief Introduction to the Staff and Student Confirmed with COVID-19 in School and Hospital of Stomatology, Wuhan University.

Patient No.^a^	Occupation	Department	Workplace	No. of Persons on Same Floor or Dept	Are There Any Close Contacts Infected?	Date of Initial Symptom
1	Doctor	Preventive dentistry	Bldg 1	10	No	Jan 23
2	Nurse	Prosthodontics	Bldg 1	51	No	Jan 28
3	Nurse	Prosthodontics	Bldg 1	51	Family members^b^	Feb 4
4	Administrator	Library	Bldg 1	2	1 family member	Jan 27
5	Nurse	Oncology surgery	Bldg 2	37	Family members	Jan 31
6	Administrator	Teaching office	Bldg 2	18	Family members^b^	Jan 29
7	MD student	Research group^c^	Bldg 3	19	1 family member	Jan 28
8	Doctor	Zhongshang clinic^d^	Satellite clinic	18	1 family member	Jan 29
9	Doctor	Yichang clinic^d^	Satellite clinic	15	1 family member	Jan 29

COVID-19, coronavirus disease 2019.

a The staff and the student confirmed with COVID-19 did not contact one another closely, and most of them had been away since January 22 or 23, 2020, because of the Chinese Spring Festival.

b Family member with COVID-19–related symptom at least 1 d earlier.

c Patient 7 is a research group member supervised by a professor. No classmates or roommates of his were reported to be infected.

d Zhongshang clinic and Yichang clinic are 2 of 16 satellite clinics of our hospital. The former, located within Wuhan city, is 4 km away from the hospital, and the latter is 300 km away and located in Yichang city. They both solely depend on air conditioners. Patients 8 and 9 are a couple. They were reunited, took a high-speed train back home on January 21, and had respiratory symptoms after 8 d.

**Figure 2.**
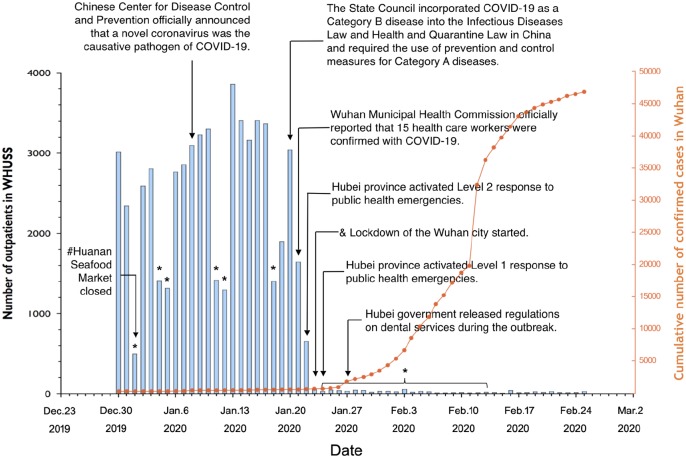
Number of outpatients treated at the School and Hospital of Stomatology, Wuhan University (WHUSS), and cumulative number of confirmed cases with coronavirus disease 2019 (COVID-19) in Wuhan city from December 30, 2019, to February 25, 2020. *Weekends and national holidays (including the extended spring festival holiday). ^#^Huanan Seafood Market was closed in Wuhan city after the majority of the earliest COVID-19 cases were linked to the Huanan Seafood Wholesale Market. ^&^Lockdown of Wuhan city started. According to the epidemic situation, the notification of city lockdown was made to stop any transportation, including airplane, train, and bus, from Wuhan city to prevent COVID-19 transmission, especially because of population movement during Chinese Spring Festival. Before January 21, WHUSS staff had only medical masks and gloves. On January 22, we started to use disposable surgical masks, N95 masks, and gowns. Goggles and protective suits were not available until January 28.

**Figure 3.**
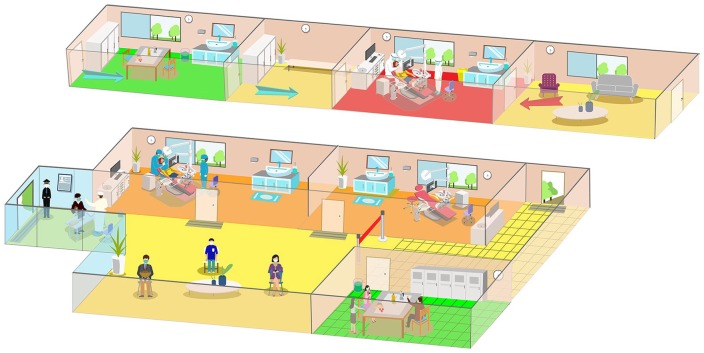
The personal protective equipment (PPE) diagram for divisions in the emergency care area at the School and Hospital of Stomatology, Wuhan University, during the coronavirus disease 2019 (COVID-19) outbreak. Yellow: triage and waiting area. Orange: dental clinic. Red: isolation clinic. Green: resting area for staff only. As shown in the diagram, our triage staff in the yellow area wear disposable surgical mask, cap, and work clothes. In the orange area, dental staff is provided with PPE, including disposable N95 masks, gloves, gowns, cap, shoe cover, and goggles or face shield. The area is disinfected once every half day. Before February 21, all the patients were treated in this area. The isolation clinic in the red area was set up on February 21. It is designed for patients who are suspected with COVID-19, who are recovering from COVID-19 (but <1 mo after they are discharged from hospital), or who need dental procedures producing droplets and/or aerosols. Separate entrances for patients (red arrow) and staff (blue arrow) are provided in the area. Dental staff should wear protective clothing besides the aforementioned PPE. In addition, the entire isolation area is disinfected immediately after the treatment is over and the patient has left. The grid area behind the red line is for staff only. Staff can have a rest in the room (green area). They are recommended to enter the room by turn and to keep wearing medical masks unless they are eating or drinking.

Despite the increasing number of confirmed cases during this period in Wuhan, we (169 staff involved in duty of dental emergency) have treated >700 patients with emergent dental treatment need since January 24 (Fig. 2), under the premise of adequate protection measures. All the dental procedures were recorded daily, and patients and their accompanying persons were requested to provide their phone number and home address in the case that either our staff or patients are suspected or confirmed with COVID-19 in the future. We have also provided consultations to >1,600 patients on our online platform since February 3. No further COVID-19 infection has been reported among our staff, which confirmed the effectiveness of our infection control measures in COVID-19 prevention within dental settings (Fig. 3).

According to the instructions from the Ministry of Education of China, all students, including those in our school, have been required to not return to school until further notification. Students are recommended to learn online after the Chinese Spring Festival on the mainland of China.

### Recommendations for Dental Practice

Interim guidance on infection prevention and control during health care is recommended when COVID-19 infection is suspected (WHO 2020a). Up to now, there has been no consensus on the provision of dental services during the epidemic of COVID-19. On the basis of our experience and relevant guidelines and research, dentists should take strict personal protection measures and avoid or minimize operations that can produce droplets or aerosols. The 4-handed technique is beneficial for controlling infection. The use of saliva ejectors with low or high volume can reduce the production of droplets and aerosols (Kohn et al. 2003; Li et al. 2004; Samaranayake and Peiris 2004).

#### Evaluation of Patients

During the outbreak of COVID-19, dental clinics are recommended to establish precheck triages to measure and record the temperature of every staff and patient as a routine procedure. Precheck staff should ask patients questions about the health status and history of contact or travel (WHO 2020a). Patients and their accompanying persons are provided with medical masks and temperature measurement once they enter our hospital. Patients with fever should be registered and referred to designated hospitals. If a patient has been to epidemic regions within the past 14 d, quarantine for at least 14 d is suggested. In areas where COVID-19 spreads, nonemergency dental practices should be postponed (Kohn et al. 2003; Li et al. 2004; Samaranayake and Peiris 2004).

It was reported that dental practice should be postponed at least 1 mo for convalescing patients with SARS (Samaranayake and Peiris 2004). It is unknown yet whether the same suggestion should be recommended for patients with COVID-19.

#### Oral Examination

Preoperative antimicrobial mouth rinse could reduce the number of microbes in the oral cavity (Kohn et al. 2003; Marui et al. 2019). Procedures that are likely to induce coughing should be avoided (if possible) or performed cautiously (WHO 2020a). Aerosol-generating procedures, such as the use of a 3-way syringe, should be minimized as much as possible. Intraoral x-ray examination is the most common radiographic technique in dental imaging; however, it can stimulate saliva secretion and coughing (Vandenberghe et al. 2010). Therefore, extraoral dental radiographies, such as panoramic radiography and cone beam CT, are appropriate alternatives during the outbreak of COVID-19.

#### Treatment of Emergency Cases

Dental emergencies can occur and exacerbate in a short period and therefore need immediate treatment. Rubber dams and high-volume saliva ejectors can help minimize aerosol or spatter in dental procedures. Furthermore, face shields and goggles are essential with use of high- or low-speed drilling with water spray (Samaranayake et al. 1989). According to our clinic experience during the outbreak, if a carious tooth is diagnosed with symptomatic irreversible pulpitis, pulp exposure could be made with chemomechanical caries removal under rubber dam isolation and a high-volume saliva ejector after local anesthesia; then, pulp devitalization can be performed to reduce the pain. The filling material can be replaced gently without a devitalizing agent later according to the manufacturer’s recommendation. We also met a patient who had a spontaneous toothache because of a cracked tooth without dental decay, and a high-speed handpiece had to be used to access cavity preparation. Given that the patient wanted to retain the tooth, she was scheduled as the last patient in the day to decrease the risk of nosocomial infection. After treatment, environmental cleaning and disinfection procedures were followed. Alternatively, patients could be treated in an isolated and well-ventilated room (Fig. 3) or negatively pressured rooms if available for suspected cases with COVID-19.

The treatment planning of tooth fracture, luxation, or avulsion is dependent on the age, the traumatic severity of dental tissue, the development of the apex, and the duration of tooth avulsion (Andersson et al. 2012; DiAngelis et al. 2012; Malmgren et al. 2012). If the tooth needs to be extracted, absorbable suture is preferred. For patients with facial soft tissue contusion, debridement and suturing should be performed. It is recommended to rinse the wound slowly and use the saliva ejector to avoid spraying. Life-threatening cases with oral and maxillofacial compound injuries should be admitted to the hospital immediately, and chest CT should be prescribed if available to exclude suspected infection because the RT-PCR test, besides time-consuming, needs a laboratory with pan-coronavirus or specific SARS-CoV-2 detection capacity.

### Recommendations for Dental Education

Education-related challenges for medical and dental schools, as well as their affiliated hospitals, are significant. It was reported that open communication among students, clinical teachers, and administrative staff would enhance mutual trust and facilitate adequate cooperation (Park et al. 2016).

On the basis of our experience with SARS and relevant highly pathogenic infectious disease, we provide a few basic recommendations for dental education during an outbreak: First, during the outbreak period, online lectures, case studies, and problem-based learning tutorials should be adopted to avoid unnecessary aggregation of people and associated risk of infection (Patil et al. 2003). Existing smart devices and applications have already made it possible for students to listen to and review lectures whenever and wherever possible. In fact, our students started online learning from February 17. Second, it is worth advocating to encourage students to engage in self-learning, make full use of online resources, and learn about the latest academic developments. Third, during this period, it is easy for students to be affected by disease-associated fear and pressure, and dental schools should be prepared to provide psychological services to those who need them (Wong et al. 2004).

With the increased knowledge of viral features, epidemiologic characteristics, clinical spectrum, and treatment, efficient strategies have been taken to prevent, control, and stop the spread of COVID-19. The infection prevention and control strategies that we have adopted are determined by the fact that we are in the center of COVID-19. Other regions should follow the recommendations from the disease control centers for infection prevention and control according to the local epidemic situation.

What should we do to improve the current infection prevention and control strategies after the epidemic? How should we respond to similar contagious diseases in the future? These are open questions in need of further discussion and research.

We must be constantly aware of infectious threats that may challenge the current infection control regimen, especially in dental practices and schools of dental medicine.

## Author Contributions

L. Meng, contributed to conception, design, data acquisition, and analysis, drafted and critically revised the manuscript; F. Hua, contributed to design and data acquisition, drafted and critically revised the manuscript; Z. Bian, contributed to conception, design, data acquisition, analysis, and interpretation, drafted and critically revised the manuscript. All authors gave final approval and agree to be accountable for all aspects of the work.

## References

[bibr1-0022034520914246] Andersson L Andreasen JO Day P Heithersay G Trope M DiAngelis AJ Kenny DJ Sigurdsson A Bourguignon C Flores MT 2012 International Association of Dental Traumatology guidelines for the management of traumatic dental injuries: 2. Avulsion of permanent teeth. Dent Traumatol. 28(2):88–96. 22409417 10.1111/j.1600-9657.2012.01125.x

[bibr2-0022034520914246] Backer JA Klinkenberg D Wallinga J 2020 Incubation period of 2019 novel coronavirus (2019-nCoV) infections among travellers from Wuhan, China, 20–28 1 2020 Euro Surveill. 25(5). doi:10.2807/1560-7917.ES.2020.2825.2805.2000062. PMC7014672 32046819

[bibr3-0022034520914246] Centers for Disease Control and Prevention. 2020 Disease burden of influenza; [accessed 2020 Feb 25]. https://www.cdc.gov/flu/about/burden/index.html.

[bibr4-0022034520914246] Chan JF Yuan S Kok KH To KK Chu H Yang J Xing F Liu J Yip CC Poon RW, et al 2020 A familial cluster of pneumonia associated with the 2019 novel coronavirus indicating person-to-person transmission: a study of a family cluster. Lancet. 395(10223):514–523. 31986261 10.1016/S0140-6736(20)30154-9 PMC7159286

[bibr5-0022034520914246] Chen H Guo J Wang C Luo F Yu X Zhang W Li J Zhao D Xu D Gong Q, et al 2020 Clinical characteristics and intrauterine vertical transmission potential of COVID-19 infection in nine pregnant women: a retrospective review of medical records. Lancet [epub ahead of print 12 Feb 2020] in press. doi:10.1016/S0140-6736(20)30360-3. PMC7159281 32151335

[bibr6-0022034520914246] Chen N Zhou M Dong X Qu J Gong F Han Y Qiu Y Wang J Liu Y Wei Y, et al 2020 Epidemiological and clinical characteristics of 99 cases of 2019 novel coronavirus pneumonia in Wuhan, China: a descriptive study. Lancet. 395(10223):507–513. 32007143 10.1016/S0140-6736(20)30211-7 PMC7135076

[bibr7-0022034520914246] Del Rio C Malani PN 2020 2019 novel coronavirus-important information for clinicians. JAMA [epub ahead of print 5 Feb 2020] in press. doi:10.1001/jama.2020.1490. 32022836

[bibr8-0022034520914246] DiAngelis AJ Andreasen JO Ebeleseder KA Kenny DJ Trope M Sigurdsson A Andersson L Bourguignon C Flores MT Hicks ML 2012 International Association of Dental Traumatology guidelines for the management of traumatic dental injuries: 1. Fractures and luxations of permanent teeth. Dent Traumatol. 28(1):2–12. 22230724 10.1111/j.1600-9657.2011.01103.x

[bibr9-0022034520914246] Guan W-J Ni Z-Y Hu Y Liang W-H Ou C-Q He J-X Liu L Shan H Lei C-L Hui DS, et al 2020 Clinical characteristics of 2019 novel coronavirus infection in China. medRxiv. doi:10.1101/2020.1102.1106.20020974.

[bibr10-0022034520914246] Holshue ML DeBolt C Lindquist S Lofy KH Wiesman J Bruce H Spitters C Ericson K Wilkerson S Tural A, et al 2020 First case of 2019 novel coronavirus in the United States. N Engl J Med [epub ahead of print 31 Jan 2020] in press. doi:10.1056/NEJMoa2001191. PMC7092802 32004427

[bibr11-0022034520914246] Huang C Wang Y Li X Ren L Zhao J Hu Y Zhang L Fan G Xu J Gu X, et al 2020 Clinical features of patients infected with 2019 novel coronavirus in Wuhan, China. Lancet. 395(10223):497–506. 31986264 10.1016/S0140-6736(20)30183-5 PMC7159299

[bibr12-0022034520914246] Kohn WG Collins AS Cleveland JL Harte JA Eklund KJ Malvitz DM; Centers for Disease Control and Prevention. 2003 Guidelines for infection control in dental health-care settings—2003. https://www.cdc.gov/mmwr/preview/mmwrhtml/rr5217a1.htm. 10.14219/jada.archive.2004.0019 14959873

[bibr13-0022034520914246] Kui L Fang YY Deng Y Liu W Wang MF Ma JP Xiao W Wang YN Zhong MH Li CH, et al 2020 Clinical characteristics of novel coronavirus cases in tertiary hospitals in Hubei province. Chin Med J (Engl) [epub ahead of print 7 Feb 2020] in press. doi:10.1097/CM1099.0000000000000744. PMC7147277 32044814

[bibr14-0022034520914246] Larson EL Early E Cloonan P Sugrue S Parides M 2000 An organizational climate intervention associated with increased handwashing and decreased nosocomial infections. Behav Med. 26(1):14–22. 10971880 10.1080/08964280009595749

[bibr15-0022034520914246] Li Q Guan X Wu P Wang X Zhou L Tong Y Ren R Leung KSM Lau EHY Wong JY, et al 2020 Early transmission dynamics in Wuhan, China, of novel coronavirus-infected pneumonia. N Engl J Med [epub ahead of print 29 Jan 2020] in press. doi:10.1056/NEJMoa2001316. PMC7121484 31995857

[bibr16-0022034520914246] Li R Leung K Sun F Samaranayake L. 2004 Severe acute respiratory syndrome (SARS) and the GDP. Part II: implications for GDPs. Br Dent J. 197(3):130–134. 15311240 10.1038/sj.bdj.4811522 PMC7091810

[bibr17-0022034520914246] Li ZY Meng LY 2020 Prevention and control of new coronavirus infection in department of stomatology. Chin J Stomatol [epub ahead of print 14 Feb 2020] in press. doi:10.3760/cma.j.issn.1002-0098.2020.0001.

[bibr18-0022034520914246] Lu R Zhao X Li J Niu P Yang B Wu H Wang W Song H Huang B Zhu N, et al 2020 Genomic characterisation and epidemiology of 2019 novel coronavirus: implications for virus origins and receptor binding. Lancet. 395(10224):565–574. 32007145 10.1016/S0140-6736(20)30251-8 PMC7159086

[bibr19-0022034520914246] Mahase E 2020 China coronavirus: WHO declares international emergency as death toll exceeds 200. BMJ. 368:m408. 10.1136/bmj.m408 32005727

[bibr20-0022034520914246] Malik YS Sircar S Bhat S Sharun K Dhama K Dadar M Tiwari R Chaicumpa W 2020 Emerging novel coronavirus (2019-nCoV)— current scenario, evolutionary perspective based on genome analysis and recent developments. Vet Q [epub ahead of print 8 Feb 2020] in press. doi:10.1080 /01652176.2020.1727993. 10.1080/01652176.2020.1727993 PMC7054940 32036774

[bibr21-0022034520914246] Malmgren B Andreasen JO Flores MT Robertson A DiAngelis AJ Andersson L Cavalleri G Cohenca N Day P Hicks ML, et al 2012 International Association of Dental Traumatology guidelines for the management of traumatic dental injuries: 3. Injuries in the primary dentition. Dent Traumatol. 28(3):174–182. 22583659 10.1111/j.1600-9657.2012.01146.x

[bibr22-0022034520914246] Marui VC Souto MLS Rovai ES Romito GA Chambrone L Pannuti CM 2019 Efficacy of preprocedural mouthrinses in the reduction of microorganisms in aerosol: a systematic review. J Am Dent Assoc. 150(12):1015–1026. 31761015 10.1016/j.adaj.2019.06.024

[bibr23-0022034520914246] National Health Commission of China. 2020a. The diagnosis and treatment protocol for novel coronavirus pneumonia (interim sixth edition) [accessed 2020 Feb 19]. http://www.gov.cn/zhengce/zhengceku/2020-02/19/content_5480948.htm.

[bibr24-0022034520914246] National Health Commission of China. 2020b. An update of novel coronavirus pneumonia outbreak as of 24:00 on February 25 [accessed 2020 Feb 26]. http://www.nhc.gov.cn/xcs/yqtb/list_gzbd.shtml.

[bibr25-0022034520914246] Park SW Jang HW Choe YH Lee KS Ahn YC Chung MJ Lee K-S Lee K Han T 2016 Avoiding student infection during a Middle East respiratory syndrome (MERS) outbreak: a single medical school experience Korean J Med Educ. 28(2):209–217. 27240893 10.3946/kjme.2016.30 PMC4951746

[bibr26-0022034520914246] Patil N Chan Y Yan H 2003 SARS and its effect on medical education in Hong Kong. Med Educ. 37(12):1127–1128. 14984121 10.1046/j.1365-2923.2003.01723.x PMC7168501

[bibr27-0022034520914246] Phelan AL Katz R Gostin LO 2020 The novel coronavirus originating in Wuhan, China: challenges for global health governance [epub ahead of print 30 Jan 2020] in press. JAMA. doi:10.1001/jama.2020.1097. 31999307

[bibr28-0022034520914246] Rothe C Schunk M Sothmann P Bretzel G Froeschl G Wallrauch C Zimmer T Thiel V Janke C Guggemos W, et al 2020 Transmission of 2019-nCoV infection from an asymptomatic contact in Germany [epub ahead of print 30 Jan 2020] in press. N Engl J Med. doi:10.1056/NEJMc2001468. PMC7120970 32003551

[bibr29-0022034520914246] Samaranayake L Reid J Evans D 1989 The efficacy of rubber dam isolation in reducing atmospheric bacterial contamination. ASDC J Dent Child. 56(6):442–444. 2681303

[bibr30-0022034520914246] Samaranayake LP Peiris M 2004 Severe acute respiratory syndrome and dentistry: a retrospective view. J Am Dent Assoc. 135(9):1292–1302. 15493394 10.14219/jada.archive.2004.0405 PMC7093872

[bibr31-0022034520914246] The Chinese Preventive Medicine Association. 2020 An update on the epidemiological characteristics of novel coronavirus pneumonia (COVID-19). Chin J Epidemiol. 41(2):139–144. 10.3760/cma.j.issn.0254-6450.2020.02.002 32057211

[bibr32-0022034520914246] Vandenberghe B Jacobs R Bosmans H 2010 Modern dental imaging: a review of the current technology and clinical applications in dental practice. Eur Radiol. 20(11):2637–2655. 20544352 10.1007/s00330-010-1836-1

[bibr33-0022034520914246] Wang D Hu B Hu C Zhu F Liu X Zhang J Wang B Xiang H Cheng Z Xiong Y, et al 2020 Clinical characteristics of 138 hospitalized patients with 2019 novel coronavirus–infected pneumonia in Wuhan, China. JAMA [epub ahead of print 7 Feb 2020] in press. doi:10.1001/jama.2020.1585. PMC7042881 32031570

[bibr34-0022034520914246] Wong JG Cheung EP Cheung V Cheung C Chan MT Chua SE McAlonan GM Tsang KW Ip MS 2004 Psychological responses to the SARS outbreak in healthcare students in Hong Kong. Med Teach. 26(7):657–659. 15763860 10.1080/01421590400006572

[bibr35-0022034520914246] World Health Organization. 2020a. Clinical management of severe acute respiratory infection when novel coronavirus (2019-nCoV) infection is suspected: interim guidance [accessed 2020 Feb 17]. https://www.who.int/publications-detail/clinical-management-of-severe-acute-respiratory-infection-when-novel-coronavirus-(ncov)-infection-is-suspected.

[bibr36-0022034520914246] World Health Organization. 2020b. Coronavirus disease 2019 (COVID-19): situation report-36 [accessed 2020 Feb 26]. https://www.who.int/docs/default-source/coronaviruse/situation-reports/20200225-sitrep-36-covid-19.pdf?sfvrsn=2791b4e0_2.

[bibr37-0022034520914246] World Health Organization. 2020c. Questions and answers on coronaviruses [accessed 2020 Feb 26]. https://www.who.int/news-room/q-a-detail/q-a-coronaviruses.

[bibr38-0022034520914246] Yang Y Lu Q Liu M Wang Y Zhang A Jalali N Dean N Longini I Halloran ME Xu B, et al 2020 Epidemiological and clinical features of the 2019 novel coronavirus outbreak in China. medRxiv. doi:10.1101/2020.1102.1110.20021675.

[bibr39-0022034520914246] Zhu H Wang L Fang C Peng S Zhang L Chang G Xia S 2020 Clinical analysis of 10 neonates born to mothers with 2019-nCoV pneumonia. Transl Pediatr. 9(1):51–60. doi:10.21037/tp.2020.02.06. 32154135 10.21037/tp.2020.02.06 PMC7036645

